# Neglected foreign body of the phalanx in a child masquerading as an osteoid osteoma: case report

**DOI:** 10.11604/pamj.2026.53.17.50879

**Published:** 2026-01-15

**Authors:** Ahmed El Mouloua, Amine El Khassoui, Tarik Salama, Elmouhtadi Aghoutane, Redouane El Fezzazi

**Affiliations:** 1 Pediatric Orthopedic and Traumatology Unit, Mother and Child Department, Mohamed VI Teaching Hospital, Cadi Ayyad University, Marrakech, Morocco,; 2 Research Laboratory for Childhood, Health and Sustainable Development, Marrakech, Morocco

**Keywords:** Foreign body, child, phalanx, hand, case report

## Abstract

Foreign body granuloma of the phalanx is an exceptionally rare entity in pediatric populations. Herein, we present a case involving a 10-year-old male patient with a granuloma of the proximal phalanx of the right index finger, initially misinterpreted as an osteoid osteoma on magnetic resonance imaging. The lesion manifested as a progressively enlarging, painful swelling over five months, with no antecedent trauma reported. Surgical exploration revealed a plant thorn embedded within an osteolytic lesion, encased by granulomatous tissue. This case underscores the importance of considering intraosseous foreign bodies in the differential diagnosis of osteoid osteoma.

## Introduction

Intraosseous foreign body (FB) granulomas are an underrecognized differential diagnosis in pediatric hand conditions. When FB penetrates, they are often localized in the lower limb, especially the foot, and are less common in the upper limb [1-3]. The bone destruction resulting from neglected FB penetration makes diagnosis difficult and can be confused with other diagnoses, including osteomyelitis and malignant bone tumors, tuberculosis, and unicameral bone cyst [4]. Plant foreign bodies are typically invisible on standard radiography, underscoring the importance of employing advanced imaging techniques, such as magnetic resonance imaging (MRI), to enhance diagnostic precision. We present an unusual case involving a foreign body within the head of the proximal phalanx in a pediatric patient, which appeared as an osteolytic cavity with a central foreign body mimicking an osteoid osteoma in a 10-year-old boy.

## Patient and observation

**Patient information:** a 10-year-old boy with an unremarkable medical history presented to the outpatient clinic for a gradually enlarging, painful swelling of the dorsolateral surface of the right index finger for 5 months. No history of trauma was recalled by the patient or the parents. No weight loss or night sweats were noted, and there was no fever.

**Timeline of the current episode:** upon presentation of the swelling, he was treated by a general practitioner as having finger cellulitis. He was given amoxicillin-clavulanate for 7 days. The swelling persisted and worsened, leading to his referral to our facility for further evaluation and specialized care.

**Clinical findings:** the physical examination showed a healthy patient. Locally, a tender mass was found in the middle of the dorsolateral aspect of the right index finger. The overlying skin was intact with no local signs of inflammation or skin break. The mobilization of the proximal interphalangeal joint was painful. Otherwise, systemic examination revealed no abnormalities.

**Figure 1:**
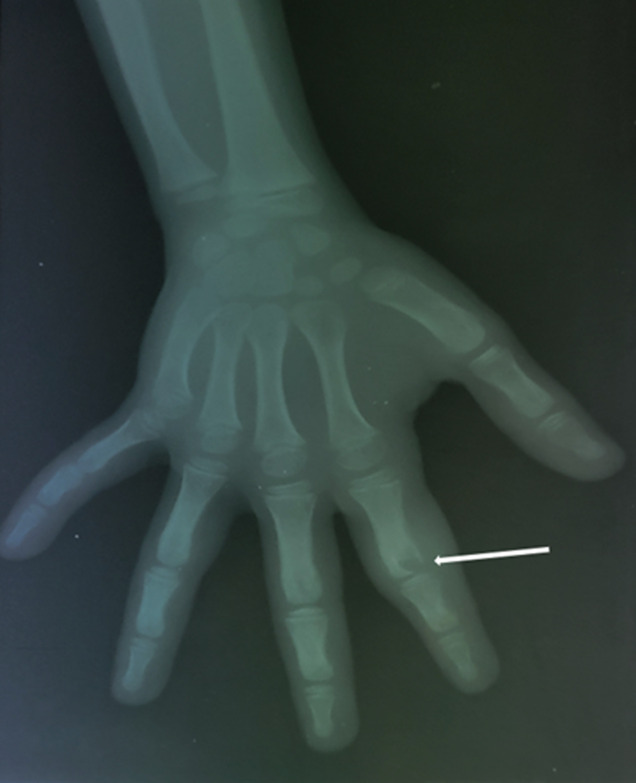
anteroposterior plain radiograph of the hand demonstrating an eccentric lytic lesion of the head and the neck of the proximal phalanx of the index finger (white arrow)

**Figure 2:**
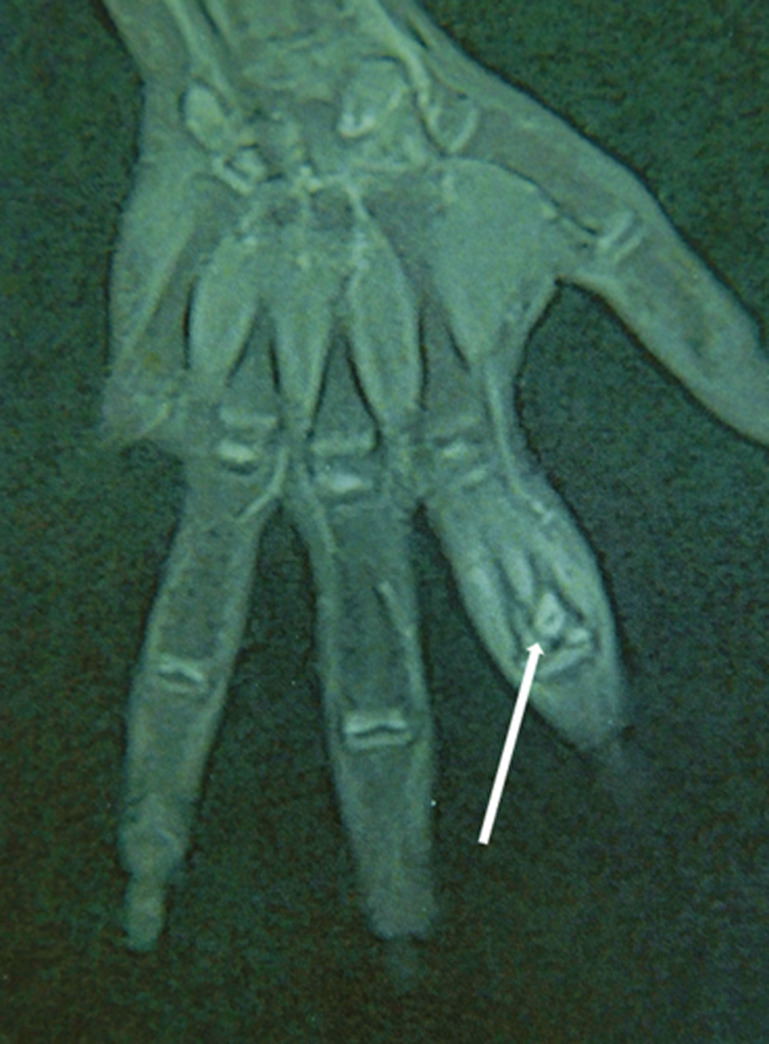
magnetic resonance imaging coronal image of the hand revealing a well-defined corticomedullary lesion in the metaphyseal-epiphyseal region with a punctate osseous center, cortical erosion at the articular surface, and associated bone marrow edema (white arrow)

**Diagnostic assessment:** laboratory investigation revealed a normal complete blood count, with slightly elevated C-reactive protein (CRP) levels at 15 mg/L (normal value < 6 mg/L), and an elevated erythrocyte sedimentation rate (ESR) of 20 mm/h (normal value < 10 mm/h). Anteroposterior and lateral radiographs of the hand showed an oblique osteolytic lesion of the cortex and the medullary space in the head and neck of the proximal phalanx of the index finger (Figure 1). An MRI of the hand showed a corticomedullary lesion in the metaphyseal-epiphyseal region of the first phalanx of the index finger, with a punctate osseous focus and significant perilesional edema, consistent with osteoid osteoma (Figure 2).

**Diagnosis:** based on the clinical history and findings on imaging, an osteoid osteoma evolving on the distal aspect of the proximal phalanx of the right index finger was suspected.

**Figure 3:**
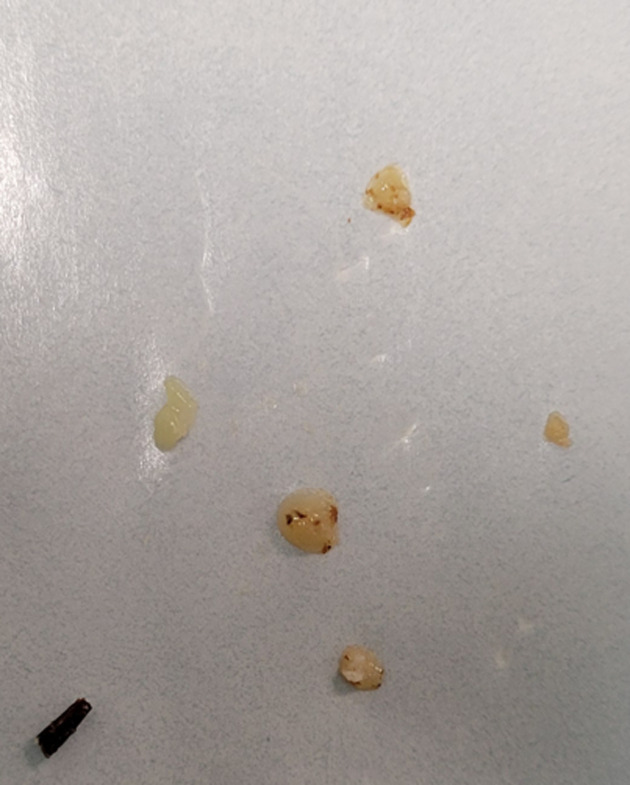
the granulation tissue and the plant thorn that were extracted during surgery

**Therapeutic interventions:** as percutaneous CT-guided radiofrequency ablation was unavailable in our hospital, we decided to perform a surgical excision. The patient was taken to the operating room under general anesthesia. A pneumatic tourniquet was applied to the arm. A curvilinear incision was made on the dorsal aspect of the finger, centered on the swelling. The extensor tendons of the index finger were then retracted. During the procedure, bony destruction of the lateral cortex of the head and neck was revealed, filled with white and swollen granulation tissue. During the removal of the tissue, a piece of a plant thorn was found in the cavity (Figure 3). A sample was sent for bacteriological and histopathologic examination. We performed a debridement and curettage, followed by irrigation of the cavity with a saline solution, without requiring any bone grafting. The incision was closed in layers with interrupted absorbable sutures. No bacteria were found in the culture of the specimen. The biopsy showed fibrous tissue centered by granulation tissue and a giant cell reaction, confirming chronic inflammation, with the presence of a foreign body consistent with a plant thorn.

**Follow-up and outcome of interventions:** the patient was discharged the following day and demonstrated complete recovery one month post-operatively with no complications. At the three-month follow-up, the cavity was observed to be partially filled on X-ray.

**Patient perspective:** when questioned further, the patient reported being pricked by a plant while playing a few months prior. Following treatment, the parents expressed their gratitude and satisfaction with the quality and outcome of the treatment.

## Discussion

Most reports in the literature indicate that the lower limb, particularly the foot, is frequently affected-likely by the penetration of FB due to its proximity to the ground and potential contact with unnoticed sharp objects [4-7]. To the best of our knowledge, this is the first pediatric case describing a foreign body that mimics an osteoid osteoma of the phalanx. The presentation of the injury is often significantly delayed across various publications, with delays from months to several years [2,6]. During this period, patients tend to forget the injury, and changes in the lesion's characteristics can occur, thereby complicating the diagnostic process and increasing the challenge for clinicians. Regarding the pathophysiology, once a foreign body (FB) penetrates the tissue, it typically triggers either an acute inflammatory response leading to phagocytosis of the FB or an infectious process that is subsequently eradicated [8]. In some cases, however, the FB becomes encapsulated by fibrous tissue, forming a granuloma. When such a granuloma is located near bone, it can induce irritation and inflammatory changes that promote osteolytic and osteoblastic activity, sometimes mimicking malignant bone tumors [3]. In our case, the localization of the FB within the center of an osteolytic lesion strongly suggested an osteoid osteoma with a central nidus represented by the FB.

Effectively differentiating FB granuloma from other clinically significant conditions, such as tumors or osteomyelitis, can be challenging. This is precisely where advanced imaging plays a crucial role, significantly enhancing diagnostic accuracy and guiding the selection of appropriate treatment. Due to their nature, FB granulomas secondary to plant thorns are often not visible on standard radiography until calcification has formed around them; They appear streaky and nodular with or without mass formation [9]. Most authors advocate the MRI as the second imaging tool to detect FB. The most typical appearance described in these reports is the hypointense signal of the foreign body on T1-weighted images surrounded with fluid rich granulation tissue or a fluid-filled cyst [3,4,6]. Besides, MRI can show the sinus tracts, soft tissue oedema, and when present, the capsule is typically hypointense on both T1- and T2-weighted images [4,7]. Despite the high sensitivity of MRI and CT scans in detecting non-metallic foreign bodies, which is approximately 97.1% and 86% respectively, detection of splinters that have been in the body for less than three days or those located near bone is not reliable by any imaging method [7,10].

## Conclusion

Although intraosseous localization of such lesions is uncommon, it is crucial to consider an organic foreign body as part of the differential diagnosis when evaluating osteoid osteoma in pediatric patients. Furthermore, any evidence of puncture or trauma from a sharp object warrants a systematic and comprehensive investigation to ensure accurate diagnosis.

## References

[1] Pushpasekaran N, Muthulingam M, Marimuthu C, Babu R, Kumar NKR (2017). Unusual Presentation of Foreign Body Granuloma of the Foot After Sharp Injury Mimicking a Malignant Lesion: a case Report. J Foot Ankle Surg.

[2] Roth S, Zaninovic M, Roth A (2017). Sponge Rubber Revealed Two Years After Penetrating Injury: A Case Report. J Foot Ankle Surg.

[3] Huang Y-M, Yang S-W, Chen C-Y, Hsu C-J, Chang W-N (2020). Residual foreign body in the foot causing chronic osteomyelitis mimicking a pseudotumor: A case report. J Int Med Res.

[4] El Bouchti I, Ait Essi F, Abkari I, Latifi M, El Hassani S (2012). Foreign body granuloma: a diagnosis not to forget. Case Rep Orthop.

[5] Gigi R, Flusser G, Kadar A, Salai M, Elias S (2017). Ochrobactrum anthropi-Caused Osteomyelitis in the Foot Mimicking a Bone Tumor: Case Report and Review of the Literature. J Foot Ankle Surg.

[6] Hassan FOA (2008). Retained toothpick causing pseudotumor of the first metatarsal: a case report and literature review. Foot Ankle Surg.

[7] Dürr HR, Stäbler A, Müller PE, Refior HJ (2001). Thorn-induced pseudotumor of the metatarsal. A case report. J Bone Joint Surg Am.

[8] Bouajina E, Harzallah L, Ghannouchi M, Hamdi I, Rammeh N, Ben Hamida R (2006). Foreign body granuloma due to unsuspected wooden splinter. Joint Bone Spine.

[9] Kim SY, Park JS, Ryu KN, Jin W, Park SY (2011). Various Tumor-Mimicking Lesions in the Musculoskeletal System: Causes and Diagnostic Approach. Korean J Radiol.

[10] Voss JO, Doll C, Raguse JD, Beck-Broichsitter B, Walter-Rittel T, Kahn J (2021). Detectability of foreign body materials using X-ray, computed tomography and magnetic resonance imaging: a phantom study. Eur J Radiol.

